# Role of melatonin in the dynamics of acute spinal cord injury in rats

**DOI:** 10.1111/jcmm.16325

**Published:** 2021-01-26

**Authors:** Jiaqi Bi, Jianxiong Shen, Chong Chen, Zheng Li, Haining Tan, Peiyu Sun, Youxi Lin

**Affiliations:** ^1^ Department of Orthopaedic Surgery Peking Union Medical College Hospital Chinese Academy of Medical Sciences and Peking Union Medical College Beijing China; ^2^ Department of Orthopaedic Surgery The First Hospital of Harbin and Harbin Institute of Technology Harbin China; ^3^ Department of Spine Surgery Orthopedics Center of Guangdong Provincial People's Hospital and Guangdong Academy of Medical Sciences Guangzhou China; ^4^ Department of Orthopedics Bejing Hospital of Traditional Chinese Medicine Beijing China

**Keywords:** Luzindole, melatonin, PI3K‐AKT1 pathway, spinal cord injury

## Abstract

Melatonin is well‐documented to have the ability of reducing nerve inflammation and scavenging free radicals. However, the therapeutic effect of melatonin on spinal cord injury has not been fully described. In this study, we assessed the effect of melatonin on T9 spinal cord injury established by Allen method in rats. Melatonin deficiency significantly delayed the recovery of sensory and motor functions in SCI rats. Treatment with melatonin significantly alleviated neuronal apoptosis and accelerated the recovery of spinal cord function. These results suggest that melatonin is effective to ameliorate spinal cord injury through inhibition of neuronal apoptosis and promotion of neuronal repair.

## 1 INTRODUCTION

Human spinal cord structure is extremely complex and sensitive to various injuries such as ischaemia, hypoxia, infection and trauma.^1^ Once the spinal cord is injured, the cell nucleus shrinks, cell body deforms, Nissl body disappears and the axon ruptures.^2^, ^3^ Neuronal necrosis releases large amounts of chemicals that act to accelerate apoptosis in surrounding neurons, is referred to as secondary damage, and induces subsequent neuroglial filling as well as impaired function.^4^, ^5^ Today, key approaches for treating spinal cord injuries include preventing cell apoptosis, reducing Nissl body dissolution, maintaining homeostasis in cellular environments and enhancing axon nutrition.^6^, ^7^, ^8^ Several substances such as growth‐associated proteins (GAPs), synaptophysin, postsynaptic density (PSD) and neurofilament (NF) proteins can promote axon regeneration and facilitate remodelling of synapses, and such affects represent core factors helping to prevent secondary damage in spinal injuries.^9^, ^10^, ^11^, ^12^

Melatonin (MT) possesses various neuroprotective effects and has been characterized at enriched levels in cerebrospinal fluid.^13^, ^14^ As an endogenous substance, MT is degraded by metabolic pathway related processes and lacks subsequent accumulation within the body.^15^ MT also was identified as effective in the treatment of SCI because of beneficial effects upon neuronal cell bodies and synapses. In particular, MT has been shown as effective in protecting mitochondria and other subcellular organelles.^16^, ^17^ Numerous studies have found that protective effects of MT upon the spinal cord were mainly manifested through scavenging of oxygen free radicals hence protecting against ischaemia and hypoxia of nerve tissue.^14^, ^18^ MT also promoted regeneration of peripheral nerve myelin sheath and stimulated various anti‐apoptosis signalling pathways.^19^, ^20^ However, the underlying mechanisms by which MT acts in such manners, and with respect to spinal cord injuries have not been fully clarified. We tried to explore the effect of melatonin on spinal cord injury, whether melatonin is a participating factor in spinal cord repair, and the impact of melatonin inhibition on spinal cord repair. Therefore, we sought to investigate functions and mechanisms of MT with respect to its use as a treatment for spinal cord injury.

## 2 MATERIALS AND METHODS

### 2.1 Animals

We purchased N = 144 9‐week‐old Sprague‐Dawley (SD) rats weighing about 250 g were from the SPF Biotechnology Company for experimentation. Rats were grown using a 12‐hour light‐dark cycle at a constant temperature of 25°C ± 5°C and with a constant atmosphere of 60‐70% humidity. Free access to water and food was provided ad‐libitum. All animal experimental protocols were reviewed and approved by the Ethics Committee of Peking Union Medical College Hospital (China).

### 2.2 Rat spinal cord injury establishment and intervention

The rat SCI model was established as previously reported.^21^ Rats were anaesthetized using 1% sodium pentobarbital (50 mg/kg). A longitudinal midline incision was made with T9 as the centre, and the spinous process and lamina of T8‐10 were exposed and rinsed with normal saline. Rats were injured by dropping a 10 g hammer from a height of 25 mm upon the T9 portion of the spinal cord followed by a manual application of a large amount of pressure upon the spinal cord for 1 minutes. Small amounts of muscle and skin were cut out, and tissues were sutured. Animals completely paralysed below the injury site were included in subsequent experiments. Urinary bladders were pressed thrice a day until the bladder regained urination reflex. The control group (CTR) was anaesthetized followed by exposure of T9 segment and suturing of the skin layer by layer.

Successfully modelled rats were randomly divided into treatment groups as follows: (i) negative control (SCI); (ii) Luzindole at 15 mg/kg, i.p. (LUZ); and (iii) melatonin, 15 mg/kg, i.p (MT). Rats in the CTR and SCI groups were injected with 1 mL of 1% ethanol saline solution. Movement and sensory recovery in all animals were examined at 1, 3, 7 and 14 days after initiation of the modelling experiment. Six rats from each group were killed for testing on each of these days upon whence T8‐T9 spinal cord segments were preserved for subsequent experiments.

### 2.3 Rehabilitation assessment

Motor function was evaluated using the BBB scale, and tail tenderness tests were performed using an electronic tenderness tester (ZS Dichuang Corporation).

### 2.4 Nissl staining

Spinal cord T8‐T9 segments were fixed in 4% formaldehyde for 4 hours and embedded in paraffin. Sections were stained in 1% cresyl violet for 10 minutes, differentiated in 95% alcohol, and subsequent Nissl bodies observed. Three 100× visual fields were randomly selected and photographed, and neurons in each visual field were counted from which averages and measures of error were determined.

### 2.5 TdT‐mediated dUTP Nick‐End Labelling (TUNEL) staining

TUNEL staining was performed following the manufacturer's instructions. Tissue sections were observed under an inverted microscope and photographed. Three 100× fields of view were randomly selected for each sample. Images were analysed using Image‐Pro 6.0 Software and spinal neurons counted. Positive cells had brown‐yellow cytoplasm and blue nuclei. Apoptosis was determined as the proportion of number of positive cells / total cells × 100.

### 2.6 RNA extraction and RT‐ qPCR analysis

Spinal cord tissues with lengths of 1 cm from centre were harvested from the points of injury and were washed with cold PBS. Spinal cord tissues were cut into small pieces and homogenized with 1 mL of Trizol reagent. Supernatant was centrifuged at 12 000 rpm for 12 minutes, and 250 μL of chloroform was added followed by mixing and additional centrifugation. To the supernatant, 0.8 volumes of isopropanol were added and samples were allowed to stand at −20°C for 15 minutes. Precipitates were washed with 75% ethanol, and DEPC water was used to help dissolve the RNA. Reverse transcription and amplification of RNA were performed following all manufacturer protocols. Primer set sequences and reaction conditions used are presented in Table 1.

**TABLE 1:** Specific primers for real‐time PCR analysis

Primer	Sequence
GAP‐43	5′‐CCAACGGAGACTGCAGAAAGC‐3′
	3′‐GTCAGCCTCGGGGTCTTCTTT‐5′
SYNAPSINⅠ	5′‐GAAGTTCTTCGGAATGGGGTC‐3′
	3′‐GTCGAACCATCTGGGCAAAC‐5′
NF‐200	5′‐TGCTCGGTCAGATTCAGGGC‐3′
	3′‐GAGCGCATAGCATCCGTGTT‐5′
PSD‐95	5′‐GGTTCCATCGTTCGCCTCTA‐3′
	3′‐GCAATGCTGAAGCCAAGTCCT‐5′
GAPDH	5′‐CTGGAGAAACCTGCCAAGTATG‐3′
	3′‐GGTGGAAGAATGGGAGTTGCT‐5′

### 2.7 Western blotting

The expression levels of NF‐200, Synapsin Ⅰ, GAP‐43, PSD‐95, PI3 Kinase p85, PTEN, p‐PDK1, PDK1, p‐AKT1, AKT, p‐NF‐κB p65, and NF‐κB p65 proteins were determined by Western blotting. Spinal cord tissue homogenates were incubated in a lysate containing protease inhibitors for 20 minutes in an ice bath.

Collected supernatant was centrifuged at 12 000 × g for 10 minutes, and protein concentrations were detected using BCA assay kits following all manufacturer protocols. Then, supernatant was diluted to a uniform concentration by adding the protein extraction reagent. Loading buffer (5X) was added to the sample at a 1:4 ratio, and samples were then boiled for 15 minutes.

Proteins were electrophoresed on polyacrylamide gels and transferred to polyvinylidene difluoride (PVDF) membranes. The PVDF membranes were blocked using TBST containing 5% non‐fat milk at room temperature for 2 hours. Primary antibody was added to the PVDF membranes which were then incubated at 4℃ overnight. Membranes were thrice washed with TBST solution, and secondary antibody was added followed by incubation at 37℃ for 2 hours. Proteins in the PVDF membranes were quantified using enhanced chemiluminescence (CW biotech) and subsequently analysed using Image Quant TL Software (GE).

### 2.8 Statistical analysis

The Statistical Package for Social Scientists (SPSS, Version 26.0, IBM) was used for data analysis. Measurements for data were expressed as the mean (M) ± standard deviation (SD). One‐way analysis of variance (ANOVA) was used to facilitate comparisons of differences between groups. LSD and S‐N‐K methods were used to facilitate comparisons among multiple groups where variances were uniform. We used Kruskal‐Wallis H tests with K independent sample tests where variances were uneven. *P* < .050 was considered as the level of statistical significance at which the null hypothesis of no differences among comparisons would be rejected.

## 3 RESULTS

### 3.1 MT accelerates the recovery of spinal cord injury in SCI rats

**FIGURE 1:**
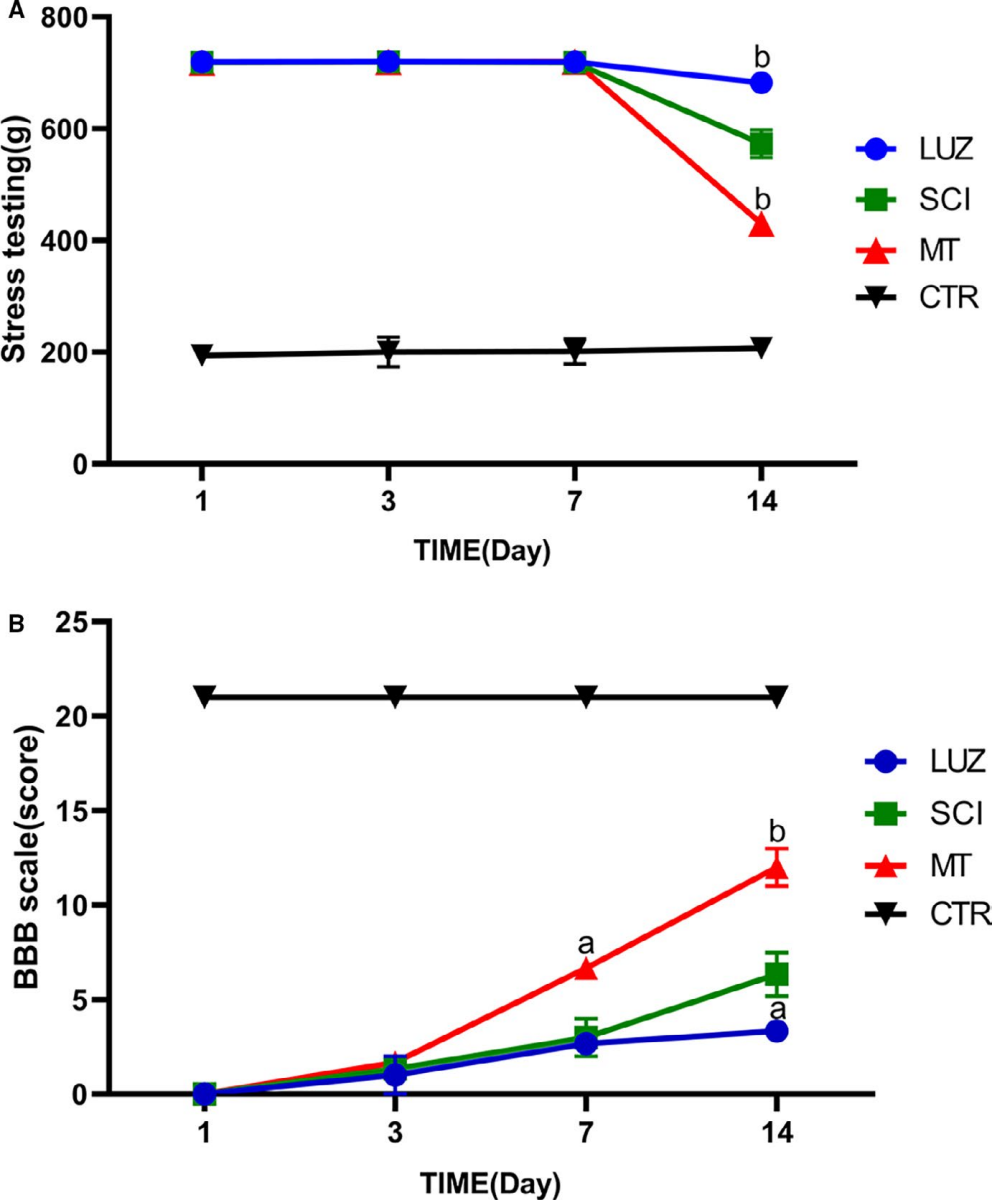
Effect of LUZ and MT on tail tenderness (A) and BBB scores (B) after SCI (N = 6). ^a^ *P* < .05, ^b^ *P* < .01. A: SCI rats' tenderness values. Following the establishment of the SCI model, tenderness values were significantly increased in each group. MT treatments significantly reduced the tenderness values of SCI rats when compared with the control model (CTR) group. In contrast, LUZ increased the tenderness value on day 14 (*P* < .01). B: BBB scores of rats. BBB scores were significantly improved in spinal cord injured rats assessed upon day 7 and day 14 following MT treatment (*P* < .05, *P* < .01). BBB scores of rats in the LUZ group were lower than in the SCI group upon day 14 (*P* < .05)

Tenderness values reflected sensory functions of rats and a decrease in tenderness indicated that sensory function was recovering. On the 14th day after SCI, tenderness values began to decrease and sensory function recovery was observed in the SCI group. Tenderness values in the MT group were reduced compared to the negative control group whereas the LUZ group showed increased tenderness values compared with the SCI group (Figure 1A).

BBB scores were used to facilitate assessment of motor function in rats. Scores were significantly improved at days 7 and 14 following MT‐based treatments. However, BBB scores of rats in the LUZ group were reduced compared with those in the negative control group upon day 14 (Figure 1B). These results indicated that MT accelerated the recovery of motor function in spinal cord injured rats, whereas LUZ delayed the recovery of motor function.

### 3.2 MT maintained Nissl body numbers and increased the stability of the internal environment

**FIGURE 2:**
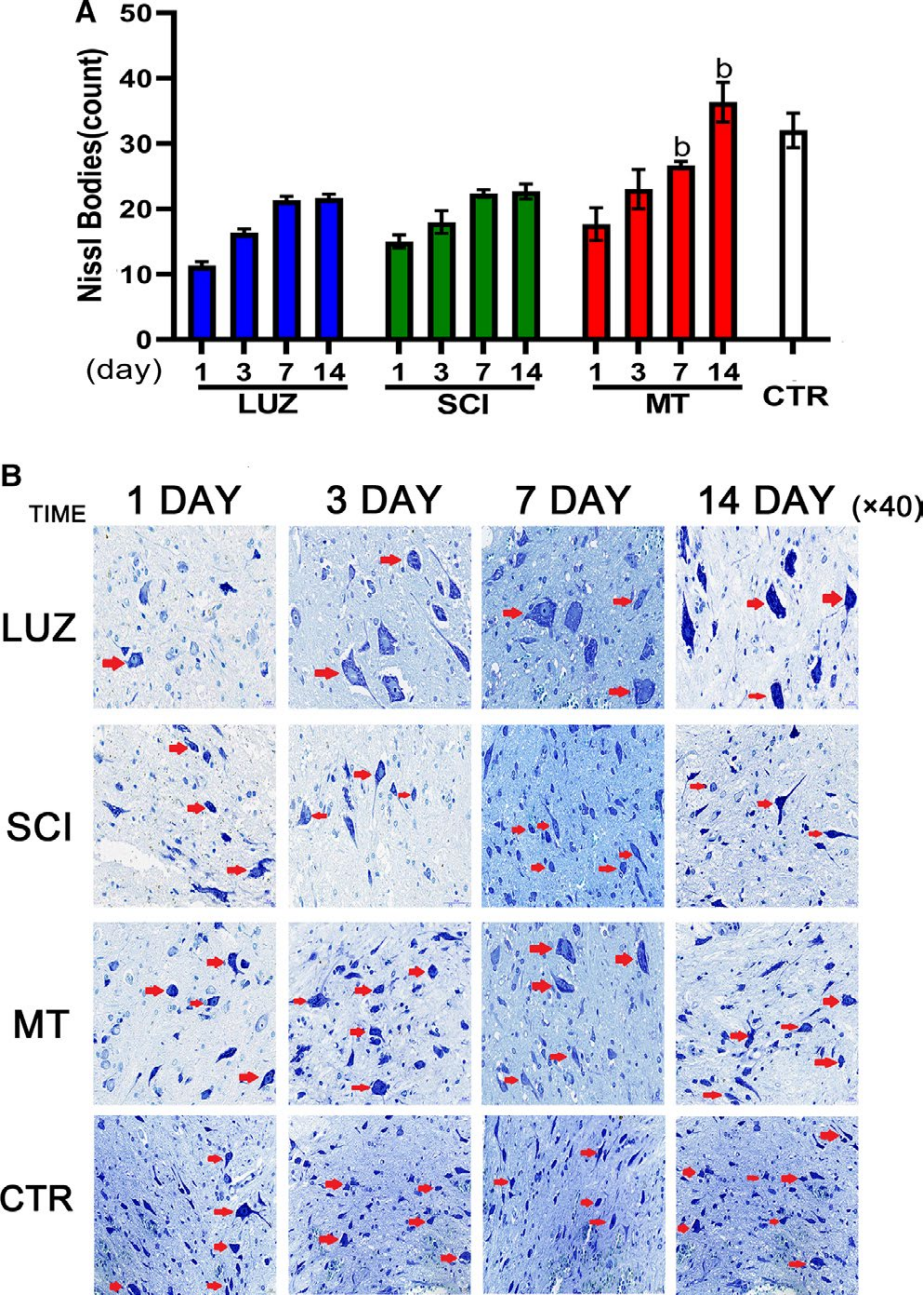
The effect of MT on Nissl bodies in SCI rats (N = 6). ^a^ *P* < .05, compared with the SCI group. A: Nissl body quantitative assessments. Upon days 7 and 14, the number of spinal Nissl bodies in the MT group was significantly higher compared with values for the SCI group (*P* < .05). B: Stained images of Nissl bodies

The number of Nissl bodies in the MT group was significantly higher compared with the SCI and LUZ groups upon days 7 and day 14 post‐induction of SCI (Figure 2A). Only small amounts of Nissl bodies were observed in each group upon the first day. Thereafter, Nissl body numbers were significantly increased in the MT group, but not in the LUZ and SCI groups (Figure 2B).

### 3.3 MT reduces neuronal apoptosis after spinal cord injury

**FIGURE 3:**
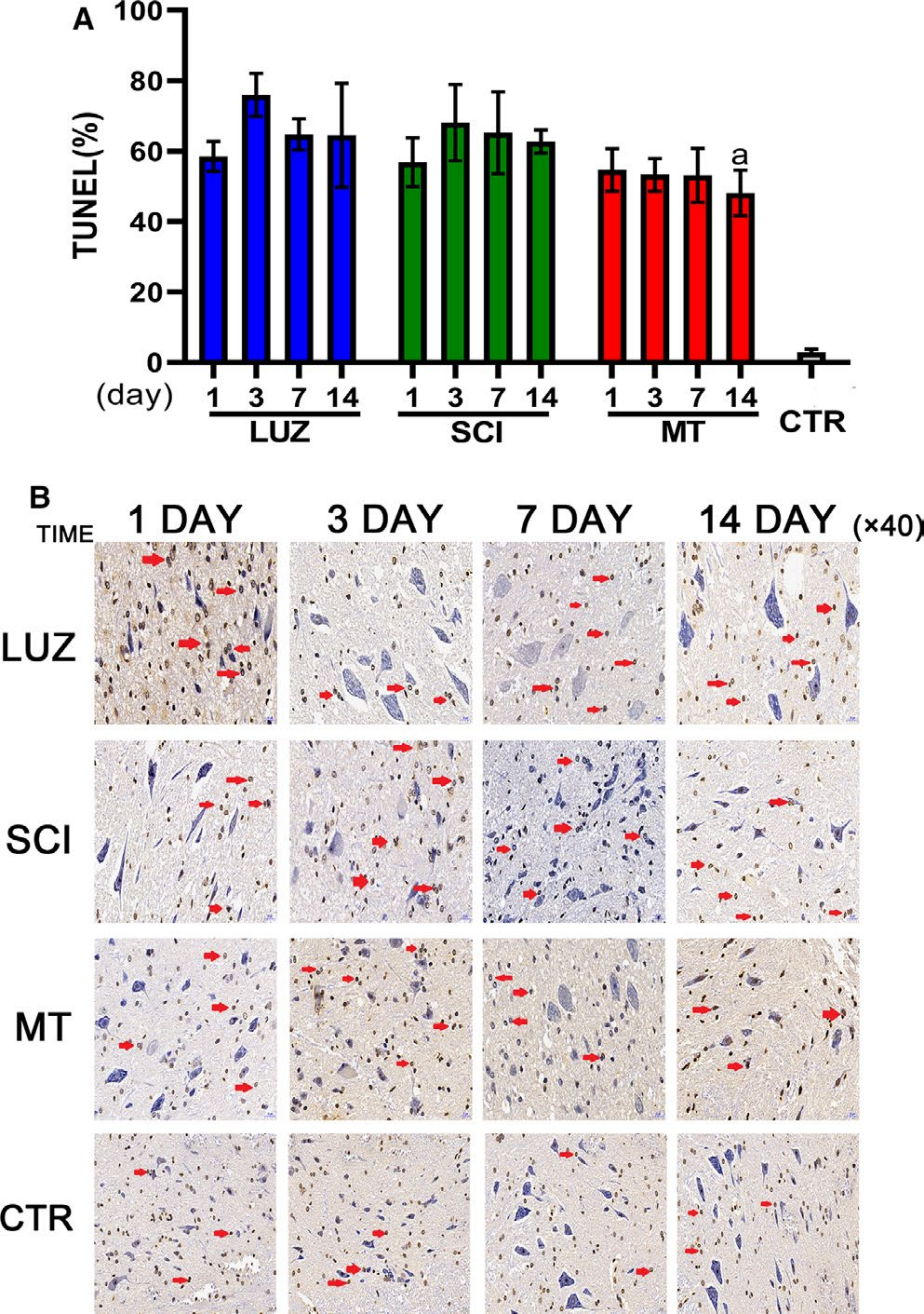
The effect of MT upon neuronal apoptosis in SCI rats (N = 6). ^a^ *P* < .05, compared with the SCI group. A: TUNEL‐positive cell percentages analyses. 14 days post‐MT treatment, TUNEL‐positive cells were significantly decreased compared with the SCI group (*P* < .05). B: Spinal cord TUNEL staining image

TUNEL staining indicated that spinal cord neurons underwent apoptosis in SCI afflicted rats. On the third day, the apoptosis rate reached its peak and then began to decline slowly. However, after 14 days of MT‐based treatments, the rate of apoptosis decreased significantly (Figure 3A,B).

### 3.4 MT activates PI3K pathway

**FIGURE 4:**
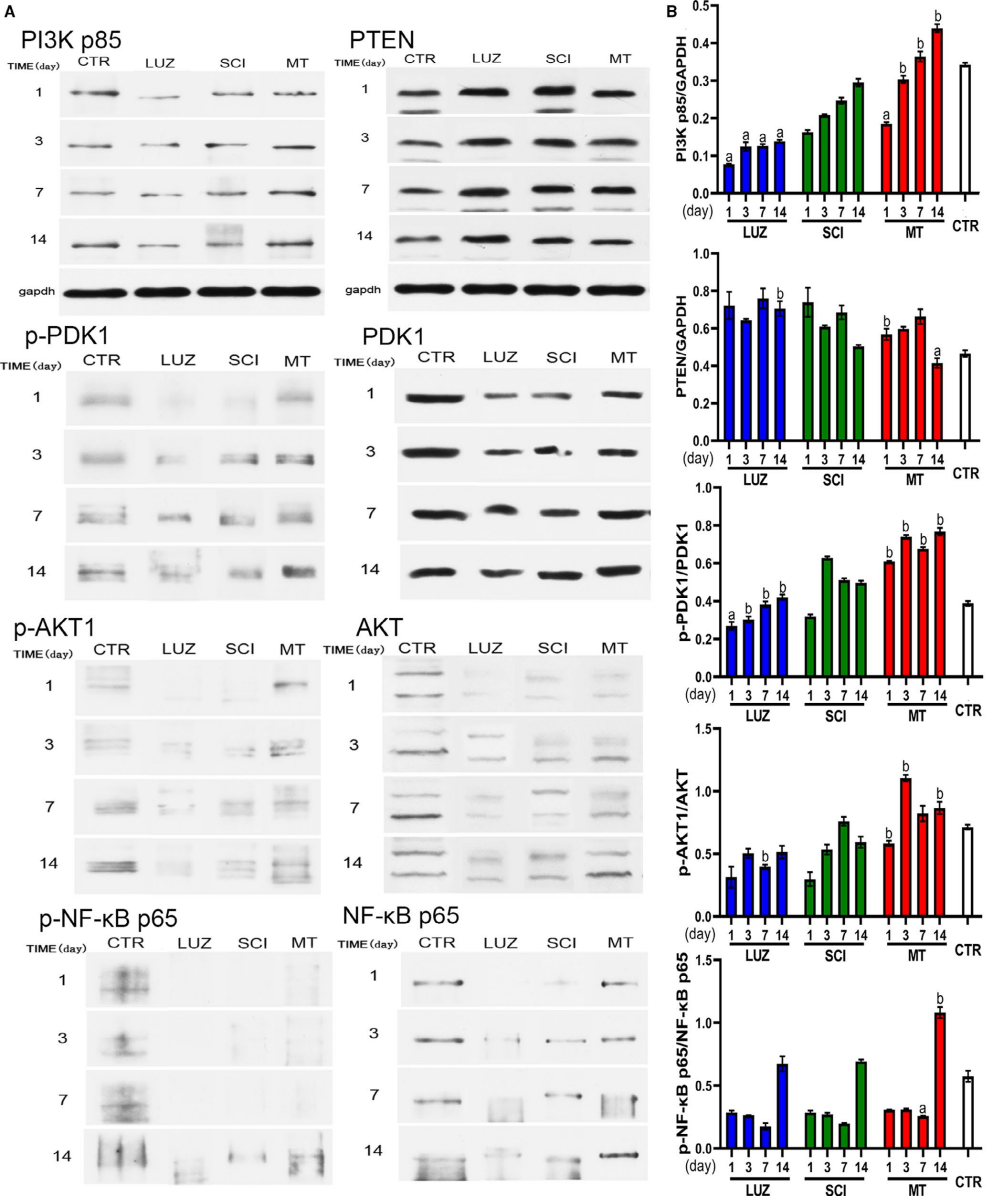
The effect of MT on protein expression levels of downstream proteins in the PI3K‐AKT1 signalling pathway in SCI rats (N = 3). ^a^ *P* < .05, ^b^ *P* < .01, compared with the SCI group. A: Immunoreactive protein bands representing PI3K p85, PTEN, PDK1, p‐PDK1, p‐AKT, AKT, p‐NF‐κB p65 and NF‐κB p65. B: Quantitative data for band intensities of PI3K p85, PTEN, p‐AKT1/AKT, p‐PDK1/PDK1, p‐NF‐κB p65 and NF‐κB p65. MT promoted PI3K protein expression and increased the phosphorylation levels of PDK1, AKT and NF‐κB p65 proteins. MT increased protein expression levels, compared with the SCI group, and the difference was significant (*P* < .05 or *P* < .01)

The expression of key factors in the PI3K pathway was determined by Western blotting analyses. MT treatments significantly increased PI3K p85 expression and reduced PTEN at each time point. Correspondingly, MT treatments increased the phosphorylation level of proteins downstream of the PI3K signalling pathway, including PDK1, AKT1 and NF‐κB p65. However, LUZ inhibited the expression of PI3K p85 and reduced the phosphorylation of the downstream proteins (Figure 4A,B). These results indicated that MT can inhibit the apoptosis of spinal neurons by activating the PI3K pathway.

### 3.5 MT increased mRNA and protein expression levels of critical proteins

**FIGURE 5:**
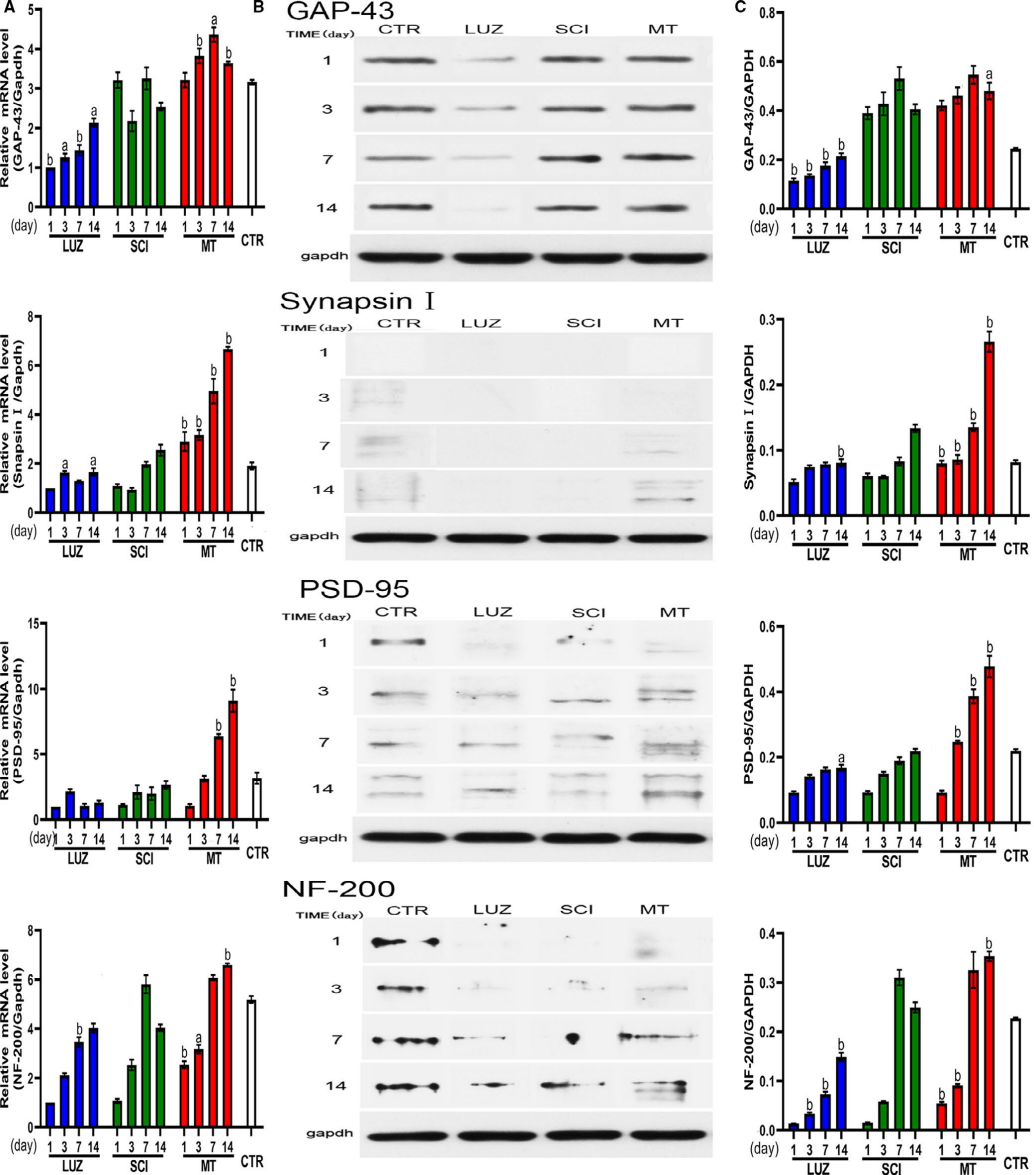
The effect of MT upon tmRNA and protein expression levels of key trophic factors in axons of spinal cord tissue in SCI rats (N = 3). ^a^ *P* < .05, ^b^ *P* < .01. A: Relative mRNA expression level of GAP‐43, Synapsin I, PSD‐95 and NF‐200. B: Immunoreactive protein bands representing GAP‐43, Synapsin I, PSD‐95 and NF‐200. C: Quantitative band intensity data for GAP‐43, Synapsin I, PSD‐95 and NF‐200. MT increased GAP‐43, Synapsin I, PSD‐95, and NF‐200, mRNA, and protein expression levels, compared with the SCI group, and the difference was significant (*P* < .05 or *P* < .01)

Increased secretion of critical proteins can help to repair damaged neurons. We found that MT treatment significantly increased mRNA and protein expression levels of GAP‐43, Synapsin I, NF‐200 and PSD‐95. Thus, MT promoted neuronal repair. However, LUZ inhibited expression of these factors and levels were lowered in comparisons to respective results for the SCI group (Figure 5A,B).

## 4 DISCUSSION

The spinal cord facilitates signal transmission between the brain and limbs. Injury to fragile aspects of the spinal cord can cut off all the information arriving or feedback from the injured part of the body and can paralyse the plane below the injured locale.^22^ Each year, about 500 000 people globally suffer from spinal cord injury, and frequently, the central neurons are in a terminally differentiated state and cannot be regenerated after damage.^2^, ^3^

The tenderness values and BBB scores were closely related to the reception of integrated pain signals upon the ventral grey matter of the spinal cord and to the transfer of motor commands to the dorsal axon. These states can generally reflect the recovery of rat sensory and motor functions.^23^, ^24^ In this study, treatments with MT stimulated the recovery of sensory and motor functions in rats, indicating that MT accelerated recovery after SCI. However, 7‐14 days were typically required to exhibit significant alleviating effects.

After spinal cord injury, Nissl bodies dissolved and released toxic substances, which promoted cell apoptosis and caused secondary injury. This is an important cause of neuronal necrosis.^25^, ^26^ Administration of MT for 7 days effectively stabilized Nissl bodies. The apoptosis rate was significantly reduced following MT treatments at 14 days. In TUNEL‐based detection assays, the protective cell bodies lacked MT in the early stages of injury and were able to cause increased necrosis, which significantly affected the recovery of nerve function.

PI3K when at rest in the cytoplasm can respond to stimuli (such as growth factor receptor, hormones, cytokines and environmental pressures) and activate different AKT (AKT1, AKT2 and AKT3) signal pathways.^27^, ^28^ AKT phosphorylation activates IκB kinase (IKKα), which is an important anti‐apoptosis process, and leads to the degradation of IκB, an inhibitor of NF‐κB.^29^ Subsequently, this causes the release of NF‐κB from the cytoplasm for nuclear translocation and activation of target genes. In this study, we found that MT treatments activated PI3K p85 and PDK1 protein production in large quantities, and inhibited PTEN protein. Therefore, MT treatments were a key factor in the initiation of the activation of the PI3K pathway, whereas LUZ inhibited PI3K pathway initiation. The levels of expression and phosphorylation of downstream proteins were detected by MT, and its inhibitors showed consistent trends from Western blotting analyses. The trends indicated that the protective effect of MT on spinal cord injuries was related to the regulation of the PI3K‐AKT1 signalling pathway. A potential drawback was that the effect of MT upon cell membranes did not start the production of downstream factor AKT1 in the early phases of our assessments and time scales measured. Thus, the PI3K pathway did not play an anti‐apoptotic role in the early stages of spinal cord injury but played a very good auxiliary role upon whence neurons began to recover.

Growth‐associated proteins are closely related to the growth and development of neurons, injury repair, axon regeneration, synaptic remodelling and so forth.^10^ GAPs can be expressed along the whole axon during neuronal development, especially in the growth cone, and can regulate signals guiding axon regeneration.^30^, ^31^ The main functional structure of the axons is a synapse. In the continuous cognition of synapses, Synapsin I and PSD‐95 are very important for facilitating the sending and receiving of synaptic signals.^9^, ^32^, ^33^ NF is an intermediate filament which exists in neurons, and whose main function is to maintain the extensibility of nerve fibre bundles and prevent them from breaking.^11^ IN addition, among the many proteins that make up NF, NF‐200 is a specific marker of NF and an important protein that maintains the shape of NF as well as also participates in the transportation of membrane proteins.^34^

In this study, GAP‐43 mRNA expression increased in MT treatments such that consequential stimulation facilitated a subsequent increase in GAP‐43, upon whence observations of starting effects upon axon repair were noted. The persistent low expression in the LUZ group confirmed that axonal repair was inhibited. Thus, MT may be a key factor influencing axonal injury repair. Synapsin I mRNA is very sensitive to MT, and actively participates in the release of neurotransmitters. LUZ can inhibit the increase of synapsin I mRNA. At initial stages of SCI, MT showed no effects upon PSD‐95 mRNA. However, significant influences were observed after the third day (*P* < .001). In the LUZ treatment group, PSD‐95 mRNA concentrations were similar to those observed in the SCI group without MT treatment and caused the loss of synaptic plasticity. This finding suggested the important role played by MT treatment in synaptic plasticity we hypothesized was supported. MT treatments showed good therapeutic effects upon NF‐200 mRNA after the third day of treatment, likely by having induced the synthesis of a large number of NF proteins in Nissl corpuscles. IN contrast, LUZ inhibited the synthesis of NF‐200 mRNA. Therefore, MT induced nerve fibre bundle synthesis, which is a process involved in the synthesis and repair of nerve fibre bundles, and thus, LUZ can inhibit the recovery of the nerve fibre bundle.

In conclusion, MT had good effects upon axon recovery and regeneration. MT deletion had an inhibitory effect upon axons. MT effected anti‐apoptotic measures of cell bodies; however, this effect only became observable in the later time steps in our data series, and LUZ did not aggravate apoptosis of cellular bodies.

## CONFLICT OF INTEREST

The authors declare no conflicts of interest.

## AUTHOR CONTRIBUTION

**jiaqi bi:** Conceptualization (equal); Data curation (lead); Writing‐original draft (lead). **Jianxiong Shen:** Conceptualization (lead); Writing‐review & editing (lead). **Chong Chen:** Conceptualization (equal); Data curation (lead); Formal analysis (lead); Visualization (equal). **zheng Li:** Conceptualization (equal); Methodology (lead); Writing‐review & editing (equal). **Haining Tan:** Formal analysis (lead); Project administration (equal); Resources (equal). **Peiyu Sun:** Resources (equal); Software (equal). **Youxi Lin:** Funding acquisition (equal); Supervision (equal); Validation (lead).

## Data Availability

The data sets used and/or analysed during the current study are available from the corresponding author on reasonable request.
